# Multimodal investigation of melanopsin retinal ganglion cells in Alzheimer's disease

**DOI:** 10.1002/acn3.51773

**Published:** 2023-04-23

**Authors:** Chiara La Morgia, Micaela Mitolo, Martina Romagnoli, Michelangelo Stanzani Maserati, Stefania Evangelisti, Maddalena De Matteis, Sabina Capellari, Claudio Bianchini, Claudia Testa, Gilles Vandewalle, Aurelia Santoro, Michele Carbonelli, Pietro D'Agati, Marco Filardi, Pietro Avanzini, Piero Barboni, Corrado Zenesini, Flavia Baccari, Rocco Liguori, Caterina Tonon, Raffaele Lodi, Valerio Carelli

**Affiliations:** ^1^ IRCCS Istituto delle Scienze Neurologiche di Bologna UOC Clinica Neurologica Bologna Italy; ^2^ Dipartimento di Scienze Biomediche e Neuromotorie Università di Bologna Bologna Italy; ^3^ IRCCS Istituto delle Scienze Neurologiche di Bologna Programma di Neurogenetica Bologna Italy; ^4^ IRCCS Istituto delle Scienze Neurologiche di Bologna Programma Neuroimmagini Funzionali e Molecolari Bologna Italy; ^5^ Dipartimento di Medicina e Chirurgia Università di Parma Parma Italy; ^6^ Dipartimento di Fisica ed Astronomia Università di Bologna Bologna Italy; ^7^ Sleep and Chronobiology Lab, GIGA‐Cyclotron Research Centre‐In Vivo Imaging University of Liège Liège Belgium; ^8^ Dipartimento di Medicina Specialistica Diagnostica e Sperimentale Università di Bologna Bologna Italy; ^9^ Alma Mater Research Institute on Global Challenges and Climate Change (Alma Climate) Università di Bologna Bologna Italy; ^10^ Dipartimento di Medicina di Base, Neuroscienze e degli Organi di Senso Università di Bari Aldo Moro Bari Italy; ^11^ Centro per le Malattie Neurodegenerative e l'Invecchiamento Cerebrale Università di Bari Aldo Moro‐ A.O. Pia Fondazione Cardinale G. Panico Tricase Italy; ^12^ CNR Istituto di Neuroscienze Parma Italy; ^13^ IRCCS San Raffaele Milan Italy; ^14^ IRCCS Istituto delle Scienze Neurologiche di Bologna Unità di Epidemiologia e Statistica Bologna Italy

## Abstract

**Objective:**

In Alzheimer's disease (AD), the presence of circadian dysfunction is well‐known and may occur early in the disease course. The melanopsin retinal ganglion cell (mRGC) system may play a relevant role in contributing to circadian dysfunction. In this study, we aimed at evaluating, through a multimodal approach, the mRGC system in AD at an early stage of disease.

**Methods:**

We included 29 mild–moderate AD (70.9 ± 11 years) and 26 (70.5 ± 8 years) control subjects. We performed an extensive neurophtalmological evaluation including optical coherence tomography with ganglion cell layer segmentation, actigraphic evaluation of the rest‐activity rhythm, chromatic pupillometry analyzed with a new data‐fitting approach, and brain functional MRI combined with light stimuli assessing the mRGC system.

**Results:**

We demonstrated a significant thinning of the infero‐temporal sector of the ganglion cell layer in AD compared to controls. Moreover, we documented by actigraphy the presence of a circadian‐impaired AD subgroup. Overall, circadian measurements worsened by age. Chromatic pupillometry evaluation highlighted the presence of a pupil‐light response reduction in the rod condition pointing to mRGC dendropathy. Finally, brain fMRI showed a reduced occipital cortex activation with blue light particularly for the sustained responses.

**Interpretation:**

Overall, the results of this multimodal innovative approach clearly document a dysfunctional mRGC system at early stages of disease as a relevant contributing factor for circadian impairment in AD providing also support to the use of light therapy in AD.

## Introduction

Circadian rhythms are characterized by a period of about 24 h, synchronized to the light–dark cycle by the projections of melanopsin retinal ganglion cells (mRGCs) to the suprachiasmatic nucleus (SCN) through the retinohypothalamic tract.^1^ These photoreceptors are a small subgroup of intrinsically photosensitive RGCs, particularly responsive to blue light and mediating mainly the non‐image forming functions of the eye, including pupillary light reflex (PLR) and sleep regulation.^2^, ^3^, ^4^ Light is the main “zeitgeber” for circadian photoentraiment. Circadian rhythm dysfunction is commonly observed in aging and is particularly prominent in Alzheimer's disease (AD)^5^, ^6^ impacting on sleep quality, cognition, mood, and longevity.^7^, ^8^ Moreover, mounting evidence indicates that the occurrence of sleep disturbances leads to ß‐amyloid deposition^9^ and that circadian dysfunction is a risk factor for dementia.^10^ The mRGC functions and their impact on circadian rhythms have been extensively investigated over the last two decades taking advantage of animal models, which allow for their genetic manipulations.^11^ However, major difficulties are encountered in evaluating their function in humans, in particular in the context of neurodegenerative disorders such as AD, due to the limited availability of in vivo investigative tools.

In vivo optical coherence tomography (OCT) and *post mortem* studies on retina and optic nerves demonstrated an age‐related loss of RGCs including also mRGCs.^12^, ^13^, ^14^ In AD patients, it has been reported the occurrence of optic neuropathy and SCN degeneration,^15^, ^16^, ^17^ as well as of mRGC loss.^18^ Moreover, ß‐amyloid deposition as extracellular plaques or affecting directly mRGCs with intracellular and axonal inclusions have been documented in AD *post mortem* retinas.^18^, ^19^

Chromatic pupillometry, allowing to assess the mRGC contribution to PLR, has been applied to evaluate this system in several neurodegenerative disorders including hereditary optic neuropathies, glaucoma, Parkinson's disease, isolated Rapid Eye Movement sleep Behaviour Disorder,^20^, ^21^ and AD.^22^, ^23^

Blue light is particularly effective in stimulating mRGCs and previous brain functional magnetic resonance (fMRI) studies conducted in controls, people suffering seasonal affective disorder (SAD) and blind subjects, demonstrated the presence of activation/deactivation of specific cerebral areas crucial for cognitive functions using light stimuli tasks designed to stimulate mRGCs.^24^, ^25^, ^26^, ^27^, ^28^, ^29^

In this study, we aimed at expanding on our previous investigation by evaluating in vivo the mRGC system in relation to circadian rhythms through a novel multimodal approach including cognitive testing, neurophthalmological assessment and OCT measures, chromatic pupillometry, actigraphy, and brain fMRI studies in a cohort of 29 mild–moderate AD patients compared to 26 controls. We also evaluated the mRGC system in 3 centenarians.

## Materials and Methods

In this study, we prospectively screened at the IRCCS Istituto delle Scienze Neurologiche di Bologna, Bologna, Italy, 33 AD patients and 42 controls. All participants signed institutional review board–approved consent forms. The local Ethical Committee approved the study (#CE 16032, Comitato Etico Interaziendale Imola‐Bologna). We also included 3 centenarians (104 years M, 101 years F and 103 years F), who underwent the neuropthalmological evaluation, pupillometry, and rest‐activity assessment by actigraphy.

For AD, the inclusion criterion was AD diagnosis at mild–moderate stage of disease^30^ (MMSE between 11 and 25) according to IWG‐2 criteria,^31^ whereas for controls the absence of cognitive dysfunction as evaluated by cognitive tests.

Exclusion criteria for patients and controls were as follows: (1) spherical or cylindrical refractive errors more than 3 or 2 diopters, (2) presence of posterior pole pathology including age‐related macular degeneration and known optic neuropathies including open‐angle glaucoma, (3) ocular pressure more than 20 mmHg, (4) severe lens opacity, (5) retinal detachment, (6) vascular retinal pathology including diabetic retinopathy, (7) shift‐workers in the last year, (8) travels through more than one time zone during the last 3 months, and (9) contraindications for MRI.

Exclusion criteria for controls included (1) excessive daytime sleepiness as assessed by the Epworth Sleepiness Scale (ESS score > 10), (2) presence of poor sleep quality as determined by the Pittsburgh Sleep Quality Index Questionnaire (PSQI index ≥7),^28^ and (3) abnormal scores (≥11) on the 21‐item Beck Depression Inventory^32^ and the State and Trait Anxiety Inventory–Y.^33^

### Neurophthalmological evaluation

Neurophthalmological evaluations included best corrected visual acuity (VA) by Snellen's chart, color vision tests (Ishihara test), slit‐lamp biomicroscopy, Goldman applanation tonometry, color fundus photography, and OCT (DRI Triton, Topcon, Tokyo, Japan). OCT protocols included the evaluation of peripapillary retinal nerve fiber (RNFL) thickness and ganglion cell layer (GCL) segmentation analysis of the macula (GCL is defined as the thickness from the inner boundary of the GCL to the outer boundary of the Inner Plexiform Layer).^23^

### Cognitive testing

All AD patients and control subjects underwent an extensive neuropsychological assessment. In particular, global cognition was evaluated by Mini Mental State Examination (MMSE)^34^ and Brief Mental Deterioration Battery (BMDB).^35^, ^36^ All tests were standardized for age and years of education in the Italian population.

### Actigraphy

Rest‐activity rhythm was evaluated using a wrist actigraph (*MotionWatch8*, CamNtech Ltd, Fenstanton, UK) worn on the non‐dominant wrist for 7 consecutive days. Subjects were instructed to wear the device continuously over the 24 h and to complete a daily sleep‐log to track: bed and raise times, daytime naps, and periods of device removal. Subjects with <5 consecutive days of recording were excluded from analysis.

All weekly participants' *MotionWatch8* files were checked for missing data. Missing values were edited as follows: missing values of <1 h were not edited; between 06:00 and 23:00 h, any missing activity data ≥1 h, but ≤3 h in total were replaced with the daily mean activity value; between 06:00 and 23:00 h, any days with missing activity data >3 h were discarded. Actigraphy recordings were analyzed using the *Motionware* software v.1.1.15a (CamNtech Ltd) to assess sleep and NPCRA (Non‐Parametric Circadian Rhythm Analysis) measures.^37^, ^38^ In particular, we included non‐parametric measures of circadian rhythms [IS, Interdaily Stability, range 0–1; IV, Intradaily Variability, range: 0–2; RA, Relative Amplitude, (M10 − L5)/(M10 + L5), range 0–1; M10, Most 10 average, activity during most 10 active hours; L5, Least 5 average, activity for least 5 active hours]. The following estimated sleep measures were also considered: SE (Sleep Efficiency, %); TST (Total Sleep Time, minutes); TIB (Time In Bed, hours); AWT (Actual Wake Time); SL (Sleep Latency, minutes); TAS (Total Activity Score); and FI (Fragmentation Index) (https://www.camntech.com/motionware‐software/). Circadian and sleep parameters were used for comparisons between AD patients and controls, and for correlations with clinical data.

Moreover, to assess group differences in circadian motor activity profile, we processed the time‐series of raw motor activity data through functional linear modeling (FLM).^39^ The seven‐day of motor activity data were averaged into a single 24‐h activity profile and fitted using a Fourier expansion model with *n* = 19 basis permutations. Groups' differences in motor activity were assessed through non‐parametric permutation *F*‐test.

### Pupillometry

Chromatic pupillometry methods are detailed in Romagnoli et al.^23^ Briefly, a Ganzfeld ColorDome full‐field stimulator (Espion V6, ColorDome Desktop Ganzfeld; Diagnosys LLC, Lowell, MA, USA) was used, and subjects were dark‐adapted for 10 min before undergoing the following conditions:

- rod‐condition: low luminance 1 s blue flash (0.001 cd/m^2^, 472 nm) under dark‐adaption;
- mRGC‐condition: high luminance 1 s blue flash (450 cd/m^2^, 472 nm) under dark‐adaption;
- cone‐condition: 1 s red flash (10 cd/m^2^, 632 nm) presented against 6 cd/m^2^ rod‐suppressing blue‐adapting field.

**Figure 1:**
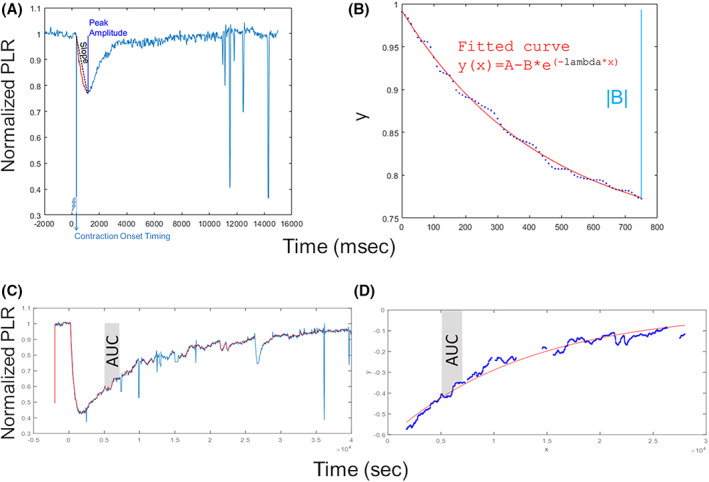
Chromatic pupillometry data analysis metrics. For rod‐condition (panels A and B), on the left, it is shown an example of normalized PLR trace (azure line) and the relative contraction onset timing and average slope calculated parameters; on the right, it is shown an example of fitted PLR curve (red curve) by using the exponential function of the form *y* = *A* – *B* * *e* ^(−lambda * *x*)^, where *A* is a constant, lambda is the constriction velocity, *x* is time in ms. For mRGC‐condition (panels C and D), on the left, it is shown an example of raw PLR trace (azure line) and of normalized PLR trace (red line); on the right, it is shown an example of exponential fitted curve (red curve) during the PLR re‐dilation phase. In both plots, the AUC during 5–7 s time interval from light‐stimulus offset is shown in gray shadow.

Data were analyzed offline using custom MATLAB scripts (MathWorks, Inc., Natick, MA, USA) as reported in details in Romagnoli et al.^23^ In addition, we complemented this quantitative report with parameters describing the PLR entity and its dynamics, tailored on individual stimulations. For the rod‐condition, we computed the contraction onset timing (the latency relative to the stimulus delivery needed for the pupil to start constriction, that is, to detach from the baseline level), the average slope (computed as the ratio between the peak amplitude and the duration of the contraction) parameters (Fig. 1A) and the parameters (see Fig. 1B) ideally fitting the contraction time course to a negative exponential curve whose equation can be summarized in *y* = *A* – *B* * *e* ^(−lambda * *x*)^.

Regarding the mRGC‐condition, the exponential dynamics of PIPR was characterized by using the MATLAB Curve FITTING Toolbox (*exp1* function, MathWorks, Inc., Natick, MA, USA) and deriving the exponential coefficient (the global rate constant of the exponential modeled PLR curve) and AUC 5–7 s (the area under the curve over a 5–7 s time interval from the light‐stimulus offset) (Fig. 1C,D).

### Brain fMRI

Brain fMRI methods can be found detailed in Evangelisti et al.^29^

Before the MR acquisitions, participants underwent a dark adaption period of 1 h and a training session for the cognitive task.

MR acquisitions were performed with a 1.5T system (GE Signa HDx 15), equipped with an 8‐channel phased array coil. The protocol included fMRI (gradient‐echoplanar sequence, slice thickness 4 mm, resolution 1.875 × 1.875 mm, repetition time TR = 3000 ms) and high‐resolution T1‐weighted images (fast spoiled gradient echo sequence, TR = 12.4 ms, TE = 5.2 ms, inversion time TI = 600 ms, voxel 1 × 1 × 1 mm^3^).

**Figure 2:**
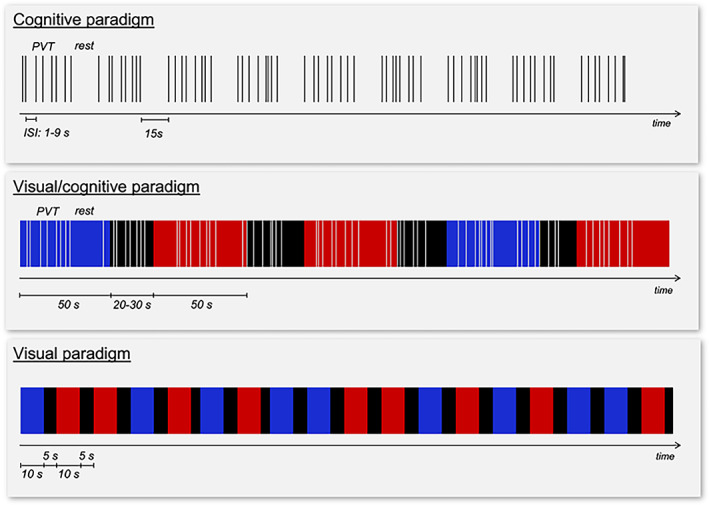
Scheme of the brain fMRI paradigms.

As for the fMRI paradigms (Fig. 2), for the *cognitive* stimulation was administrated the auditory Psychomotor Vigilance Task (PVT).^40^ It consisted in the presentation of a series of sounds separated by intervals between 1 and 9 s, and participants were required to respond to each sound as quickly as possible by pressing a button. Overall, 10 blocks were presented, separated by rest interval of 15 s, for a total duration of ~10 min. This paradigm was aimed to evaluate cognitive brain responses of sustained attention. The task was also acquired in combination with a simultaneous light stimulation (*visual cognitive* paradigm, duration ~20 min) consisting of an alternation of blue and red illumination periods of 50 s and darkness (duration from 20 to 30 s). This paradigm allowed the evaluation of the effects on brain responses of the interactions between light stimulation and cognitive task. Finally, the *pure visual* paradigm was meant to investigate the possible role of mRGCs in a pure visual setting. Participants were exposed to blue or red lights for periods of 10 s separated with 5 s of darkness, with a random color alternation, for a total duration of ~10 min.

Monochromatic light stimulation was achieved with a metal‐free purpose‐built optic fiber combined with narrow (FWHM 10 nm) interference band‐pass filters (blue 480 nm, red 620 nm).

The image analyses were carried out using the FSL software (https://fsl.fmrib.ox.ac.uk/fsl).

After image pre‐processing, single subject brain responses were estimated with a General Linear Model. For each paradigm, the events of interest were the following: for the “*Cognitive*” paradigm, the stimuli of the PVT paradigm were described with stick functions; for the “*Visual*” paradigm, the periods of lighting with blue and red were modeled with blocks and the switching on/off of the lighting with sticks; for the “*Visual/Cognitive*” paradigm, the stimuli of the PVT paradigm were described as sticks and the periods of blue and red were modeled with blocks. The activation maps were linearly registered to the T1‐weighted structural images, and then non‐linearly aligned to the standard space of the MNI template.

### Statistical analysis

Between group analyses were performed to compare demographics, OCT, actigraphy, and pupillometry outcomes. Individual patient's data were compared by using chi‐square, independent sample *t*‐, and the Mann–Whitney *U*‐tests. Normality of all continuous variables was checked by using Shapiro–Wilk and Kolmogorov–Smirnov test. Spearman correlation coefficients were computed to measure the degree of association between OCT/actigraphy/pupillometry findings or clinical variables (disease duration, MMSEc), Bonferroni's correction method was used, and two‐sided *p*‐values are presented. Statistical analyses were carried out using R software (version 4.0.0) and IBM SPSS Statistics for Windows, version 20.0 (IBM Corp., Armonk, N.Y., USA) software.

### OCT

Between groups (AD versus controls) analyses to compare OCT outcomes were performed by following the “all‐eyes approach”.^41^ OCT variables were compared by using linear mixed‐effect model (LMM) with the visual outcome as the dependent variable, the group as independent variable, under a compound symmetry covariance structure and with a patient random effect, while adjusting for age‐decade and gender.^42^ Alternatively, for OCT outcomes with skewed distribution, we applied the Clustered Wilcoxon rank sum test using Rosner‐Glynn‐Lee method.^43^ Moreover, *p*‐value for Group×Age decade interaction term was calculated with log‐likelihood ratio test (LR test) comparing nested LMMs with and without the interaction term, and stratified *β* coefficients (95% confidence interval, 95% CI) for the variables turning out to be effect modifiers (interaction term *p*‐value <0.20).

### Actigraphy

Comparison of actigraphy measures between AD and control groups were performed through independent *t*‐ and Mann–Whitney *U‐* tests. Moreover, *p*‐value for Group × Age decade interaction term was calculated with LR test comparing nested linear models with and without the interaction term, and stratified *β* coefficients (95% CI) for the variables turning out to be effect modifiers (interaction term *p*‐value <0.20).

### Pupillometry

For all conditions (rod/mRGC/cone), pupillometry metrics were obtained by their individual average values. Statistical analyses included Wilcoxon signed‐rank or independent t‐tests and aimed to compare variables among groups (AD/controls).

### Brain fMRI

Group comparisons were performed by non‐parametric permutation statistics. For the visual paradigm, comparisons were made within the primary visual cortex (based on the Juelich histological atlas^44^), while for cognitive and visual/cognitive ones, comparisons were made within the entire gray matter. Statistical inferences were made from statistical maps corrected for multiple comparisons (FWE, Family Wise Error, *p* < 0.05).

Demographics of participants recruited for fMRI examinations were compared between the two groups using non‐parametric testing. To compare the performance in the cognitive task during MRI acquisitions in different light conditions, a Friedman test was performed.

The fMRI results were correlated, by non‐parametric Spearman test, with the data related to the neuropsychological evaluation, with the measurements of RNFL, with pupillometry and actigraphic measures.

## Results

**Figure 3:**
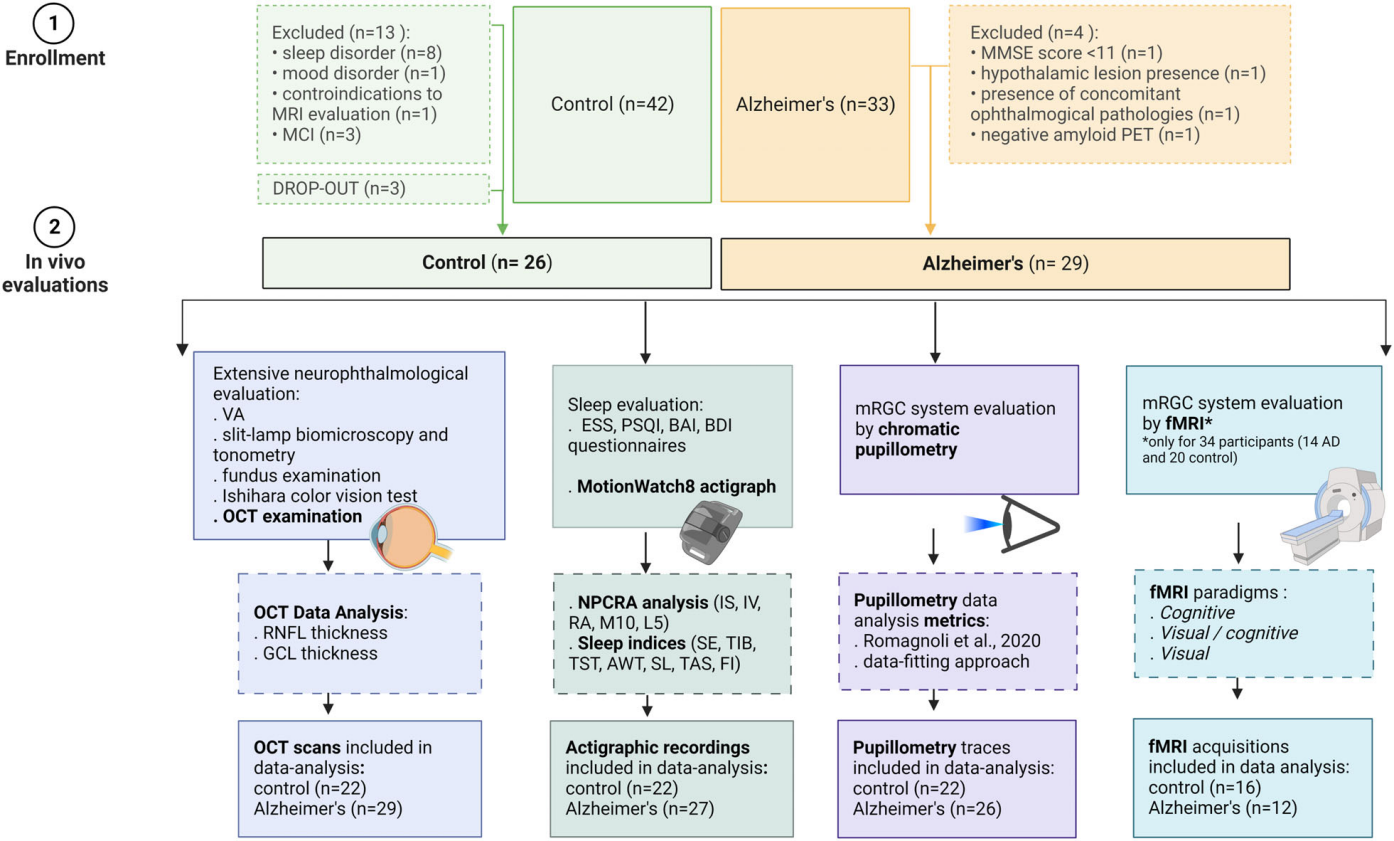
Study‐flow diagram. The image shows the study‐flow diagram of the study indicating the final cohort included and the evaluations performed for both AD and controls.

According to inclusion and exclusion criteria, 13/42 controls and 4/33 AD were excluded and 3 controls dropped‐out after the screening visit (Fig. 3). Ultimately, 26 controls and 29 AD were included in the study.

Demographic and main clinical data of the examined subjects are reported in Table 1.

**Table 1:** Sociodemographic data.

	Controls	Alzheimer's	*p*‐value
*N*	26 (47.3%)	29 (52.7%)	
Gender			
Male	9 (34.6%)	15 (51.7%)	0.2
Female	17 (65.4%)	14 (48.3%)	
Age (mean ± SD), years	70.5 ± 8	70.9 ± 11	0.89
Age decade			
50–59 years	3 (11.5%)	6 (20.7%)	0.32
60–69 years	9 (34.6%)	4 (13.8%)	
70–79 years	9 (34.6%)	12 (41.4%)	
≥80 years	5 (19.3%)	7 (24.1%)	
MMSEc	27.9 ± 1.4	20.2 ± 4.2	*p* < 0.0001
Disease duration, years	–	3.9 ± 2.8	–

### Optical coherence tomography evaluation

According to exclusion criteria, OCT scans of 4 controls were excluded from analysis. Therefore, OCT data of control (*n* = 22) and AD (*n* = 29) subjects are reported in Table 2. RNFL thickness was not different between AD and controls. Only the GCL thickness of the IT sector was significantly reduced in AD patients compared to controls (*p* = 0.034). All other GCL measurements were not significantly different between groups.

**Table 2:** Descriptive statistics for OCT data.

	Controls	Alzheimer's	*p*‐value^1^	*p*‐value^2^
*N*, subjects	22 (43.1%)	29 (56.9%)		
*N*, eyes	40	51		
*n*, Right eyes	18	27		
*n*, Left eyes	22	24		
RNFL, AVG μm	105.4 ± 2.1	106.6 ± 1.8	0.99^a^	0.94
RNFL, T μm	77.8 ± 1.95	76.2 ± 1.7	0.16^a^	0.14
RNFL, S μm	127.9 ± 2.98	131.4 ± 2.6	0.32^a^	0.89
RNFL, N μm	82.1 ± 3.0	83.4 ± 2.6	0.13^b^	0.81
RNFL, I μm	134 ± 3.3	135.4 ± 2.9	0.92^a^	0.82
GCL, AVG μm	70.4 ± 1.15	69.4 ± 0.95	0.40^a^	0.16
GCL, ST μm	71 ± 1.1	69.3 ± 0.9	0.10^a^	0.20
GCL, S μm	69.6 ± 1.2	68.8 ± 0.97	0.51^a^	0.035
GCL, SN μm	72 ± 1.3	71.8 ± 1.1	0.74^a^	0.15
GCL, IN μm	71.1 ± 1.3	70.1 ± 1.1	0.52^a^	0.48
GCL, I μm	66.9 ± 1.2	66 ± 1.0	0.13^b^	0.30
GCL, IT μm	72 ± 1.3	70.6 ± 1.1	0.034^b^	0.55

^1^ *p*‐value referred to: ^a^ “Group” predictor of linear mixed‐effect model (LMM, maximum likelihood method, random intercept, compound symmetry) for OCT variables normally distributed; ^b^Comparison of OCT variables not‐normally distributed by using Clustered Wilcoxon rank sum test, Rosner–Glynn–Lee method.

^2^ *p*‐value referred to likelihood‐ratio test comparing the goodness of fit of two competing LMM models (with and without interaction term Group × Age decade) for each OCT parameters; variables highlighting a significant effect modifier are those with *p*‐value for interaction < 0.20.

**Figure 4:**
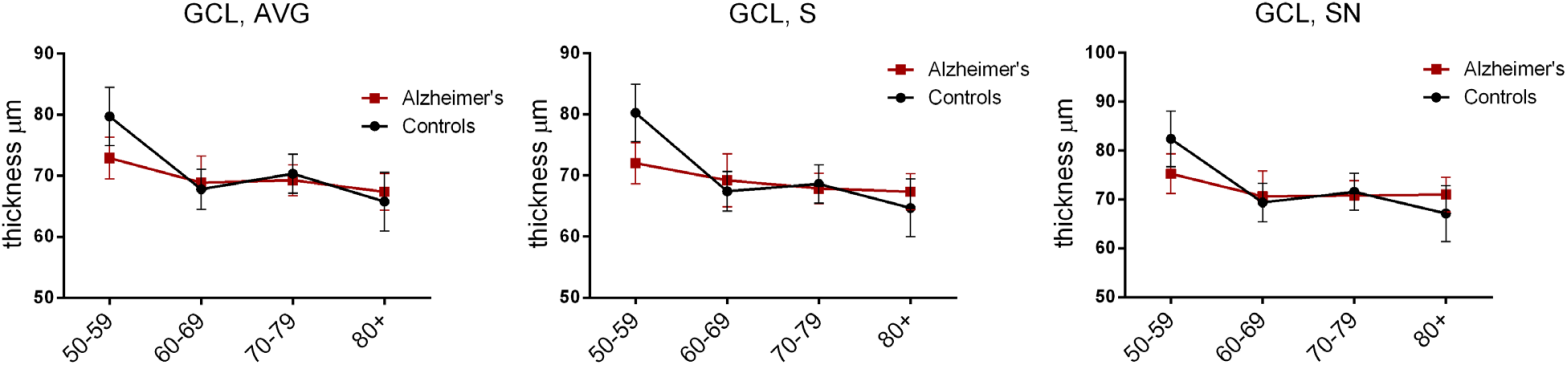
Optical coherence tomography evaluation results. Figure shows plots of estimated marginal means of GCL thickness sectors [average (AVG), superior (S), supero‐nasal (SN)] from LMM in controls and AD for each age decade and error bars representing SE. In particular, *β* coefficients for the control group: GCL, AVG: *β*_60–69 years_ = −12.2 (95% CI = −17.6 to −6.7); *β*_70–79 years_ = −9.6 (95% CI = −14.8 to −4.3); *β*_≥80 years_ = −14 (95% CI = −20 to −8). GCL, S: *β*_60–69 years_ = −12.7 (95% CI = −18.2 to −7.2); *β*_70–79 years_ = −11.5 (95% CI = −16.8 to −6.2); *β*_≥80 years_ = −15.5 (95% CI = −21.6 to −9.4). GCL, SN: *β*_60–69 years_ = −13.3 (95% CI = −19.6 to −7); *β*_70–79 years_ = −11 (95% CI = −17.1 to −5); *β*_≥80 years_ = −15.2 (95% CI = −22.2 to −8.3). *β* coefficients for AD group: AVG, GCL: *β*_60–69 years_ = −4.1 (95% CI = −10.1 to 1.8); *β*_70–79 years_ = −3.5 (95% CI = −8.2 to 1.1); *β*_≥80 years_ = 5.4 (95% CI = −10.5 to −0.3). AVG, average; GCL, Ganglion cell layer; S, superior; SN, supero‐nasal.

The Group × Age decade interaction term analysis revealed significant differences between groups for the following OCT parameters: temporal RNFL thickness, and average, superior and supero‐nasal GCL thickness (Table 2). Specifically, looking at *β* coefficients stratified by group (AD/control), we found for controls a significant decrease for the average, superior and supero‐nasal GCL thickness for the 60–69, 70–79, and over 80 decades if compared to the decade 50–59, while in AD we observed a significant decrease only for the average GCL thickness in the comparison between the decade over 80 and 50–59 (Fig. 4).

### Actigraphy

**Figure 5:**
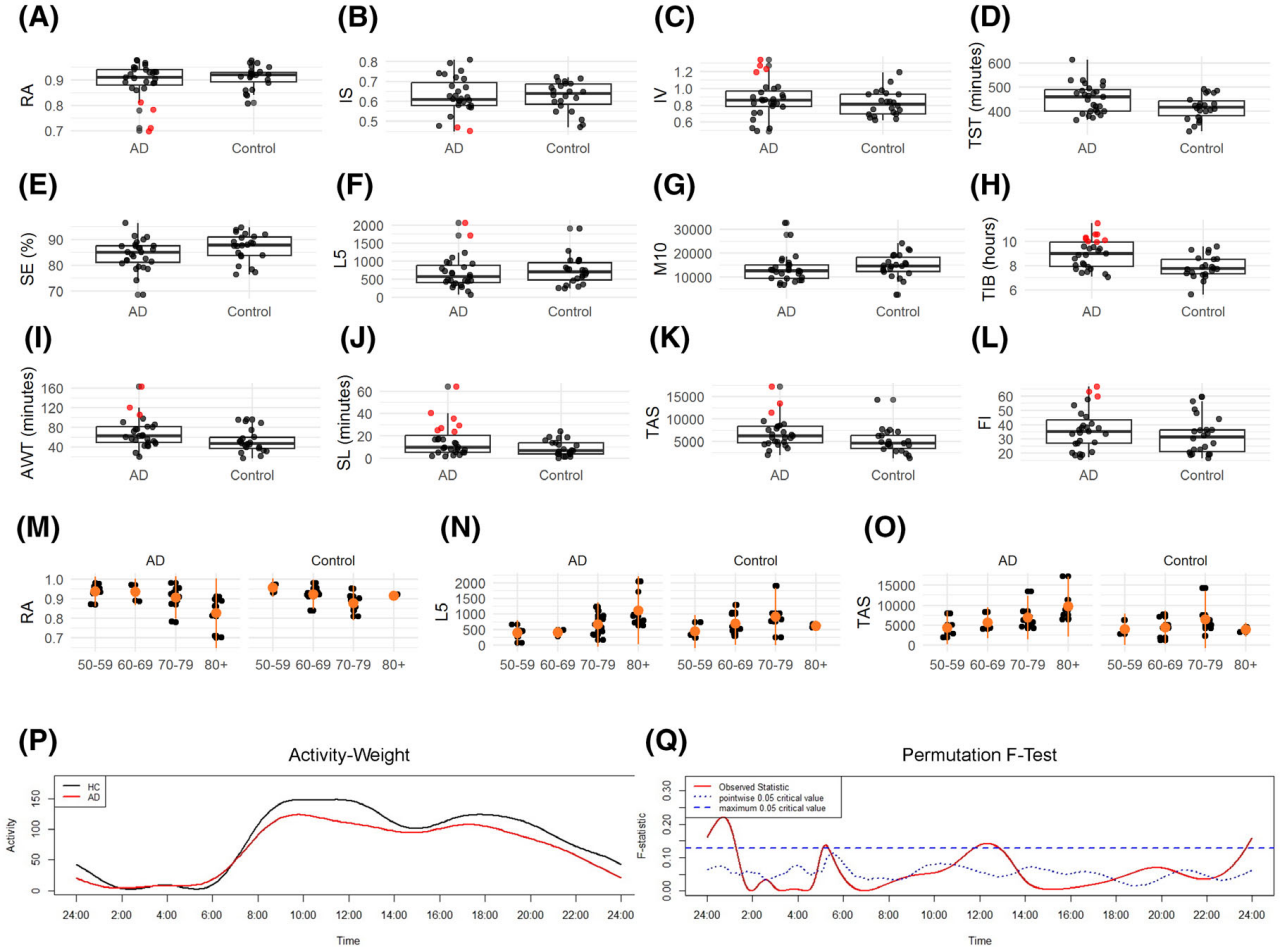
Actigraphy results. Panels A–L show boxplots of actigraphic measures for each comparison group (AD, Alzheimer's disease/control) with the horizontal line represents the median value and red plots corresponding to patients resulted as circadian‐impaired [AD cases with circadian values >2 SD from the mean of controls, as previously proposed by Hatfield et al.^45^]. Panels M–O show scatterplots of RA, L5, TAS actigraphic measures relative to AD and controls across age decades: 50–59 years; 60–69 years; 70–79 years, ≥80 years. In particular, *β* coefficients for the controls: RA: *β*_60–69 years_ = −0.05 (95% CI = −0.1 to −0.01); *β*_70–79 years_ = −0.09 (95% CI = −0.1 to −0.05); *β*_≥80 years_ = −0.05 (95% CI = −0.11 to 0.01). L5: *β*_60–69 years_ = 451 (95% CI = −12 to 914); *β*_70–79 years_ = 611 (95% CI = 153–1069); *β*_≥80 years_ = 259 (95% CI = −349 to 867). TAS: *β*_60–69 years_ = 1843 (95% CI = −1597 to 5283); *β*_70–79 years_ = 3362 (95% CI = −41 to 6765); *β*_≥80 years_ = 405 (95% CI = −4110 to 4920). *β* coefficients for AD: RA: *β*_60–69 years_ = 0.01 (95% CI = −0.07 to 0.09); *β*_70–79 years_ = −0.04 (95% CI = −0.1 to 0.03); *β*_≥80 years_ = −0.12 (95% CI = −0.2 to −0.05). L5: *β*_60–69 years_ = −36 (95% CI = −548 to 476); *β*_70–79 years_ = 295 (95% CI = −109 to 698); *β*_≥80 years_ = 779 (95% CI = 329–1228). TAS: *β*_60–69 years_ = 799 (95% CI = −3057 to 4655); *β*_70–79 years_ = 2791 (95% CI = −248 to 5830); *β*_≥80 years_ = 5850 (95% CI = 2467–9234). Panel P: Circadian motor activity profile of AD patients (Red) and controls (Black). Panel Q: Results of the non‐parametric permutation *F*‐test. Significant differences are detected when the red solid line (i.e., observed statistic) is above the blue dotted line (i.e., the point‐wise test of significance at *α* = 0.05) or the blue dashed line (i.e., the global test of significance at *α* = 0.05, more conservative). AWT, actual wake time; FI, fragmentation index; IS, interdaily stability; IV, intradaily variability; L5, least 5 average, activity for least 5 active hours; M10, most 10 average, activity during most 10 active hours; RA, relative amplitude; SE, sleep efficiency; SL, sleep latency; TAS, total activity score; TIB, time in bed; TST, total sleep time.

Actigraphic data were available for 22 controls and 27 AD. We found a significant difference between controls and AD for the following actigraphic parameters: TST (*p* = 0.01), TIB (*p* = 0.001), AWT (*p* = 0.04), SL (*p* = 0.04) and TAS (*p* = 0.04) (Fig. 5, panels D, H–K).

Overall, even if there were no significant differences, AD patients showed higher variability for circadian measures compared to controls, with some subjects clearly distinct from the control group. The circadian‐impaired patients were defined as cases with circadian values >2 standard deviations (SD) from the mean of controls, as previously proposed^45^ (Fig. 5, red plots in panels A–L).

Considering the Group × Age decade interaction term analysis, we found a significant difference between groups for RA, L5, and TAS (Fig. 5, panels M–O). Specifically, if we look at the single decades, for RA a significant difference was evident in the comparison between the decades 60–69 and 70–79 versus 50–59 in controls and between the over 80 versus 50–59 in AD (Fig. 5M), for L5 in the comparison between 70–79 versus 50–59 in controls and over 80 versus 50–59 in AD (Fig. 5N), for TAS in the comparison between over 80 versus 50–59 in AD (Fig. 5O).

Moreover, AD patients presented significantly lower motor activity from 24:00 to 2:00 (global test of significance), from 11:00 to 14:00 (point‐wise test of significance), and from 18:00 to 21:00 (point‐wise test of significance) compared to controls (Fig. 5, panels P,Q).

We also evaluated the actigraphic recordings of the 3 centenarians which did not significantly differ from the controls in the decade 80–90 (data not shown). Furthermore, SE was significantly higher in the centenarians compared to AD in the group over 80s (*p* = 0.03), as well as AWS (*p* = 0.02) and TAS (*p* = 0.005) were significantly reduced in centenarians compared to AD in the group over 80s.

### Pupillometry

Pupillometry recordings included in the final analysis, according to inclusion/exclusion criteria, were available for 22 controls and 26 AD. Under rod‐condition, AD patients showed a significantly delayed onset of transient PLR response [contraction onset timing (ms): AD = 362 (50); Control = 323 (24); *p* = 0.001] as well as a significantly lower average slope [average slope (1/ms): AD = 2.8 × 10^−4^ (1 × 10^−4^); Control = 3.4 × 10^−4^ (5.4 × 10^−5^); *p* = 0.035], and also a significantly reduced |B| [|B|: AD = 0.24 (0.08); Control = 0.29 (0.08); *p* = 0.034] indicative of a lower exponential decay amplitude. Under mRGC‐condition, no significant differences were observed among AD and control groups. Finally, we evaluated the PLR in the 3 centenarians demonstrating a significant reduction only for the PIPR parameter (*p* = 0.008) in the comparison with controls in the 80–90 decade (data not shown).

### Correlations between actigraphic and pupillometric variables

For each group (control/AD), a Spearman correlation coefficient was computed to assess the relationship between actigraphy/pupillometry/clinical variables (disease duration and MMSEc), the latter only for AD group.

In controls, there was a strong, negative correlation between RA and contraction onset timing (rod‐condition) with *r* = −0.84 (CI 95% = −0.94 to −0.60, *p*_adjusted_ = 0.001), and a strong, positive correlation between M10 and the slope (rod‐condition) of PLR with *r* = 0.83 (CI 95% = 0.57–0.94, *p*_adjusted_ = 0.002). The same bivariate relationships were lost in AD group (data not shown). In AD, there was only a moderate, negative correlation between TIB and |B| with *r* = −0.65 (CI 95% = −0.84 to −0.32, *p*_adjusted_ = 0.048) (Supplementary Materials).

### Functional MRI

Among the 55 subjects enrolled in the study, 7 participants were not available to perform the fMRI. From the remaining 48, 14 subjects were not included because of claustrofobia or not‐compatible MRI implanted devices. For 34 participants (14 AD and 20 controls), the fMRI acquisitions were completed but 6 were excluded for suboptimal image quality.

The final dataset for fMRI studies consists of 12 AD (mean age 66.8 ± 10.0 years, 8 males, all right‐handed and with a mean MMSEc score of 22.4 ± 5.5) and 16 controls (mean age 72.1 ± 8.2 years, 5 males, all right‐handed and with a mean MMSEc score of 29.0 ± 0.9). The two groups differed significantly, as expected, for the cognitive level assessed with MMSEc (*p*‐value 0.001), while there was no statistically significant difference for age nor gender (*p* = 0.271 and *p* = 0.141, respectively).

### Visual stimulation

**Figure 6:**
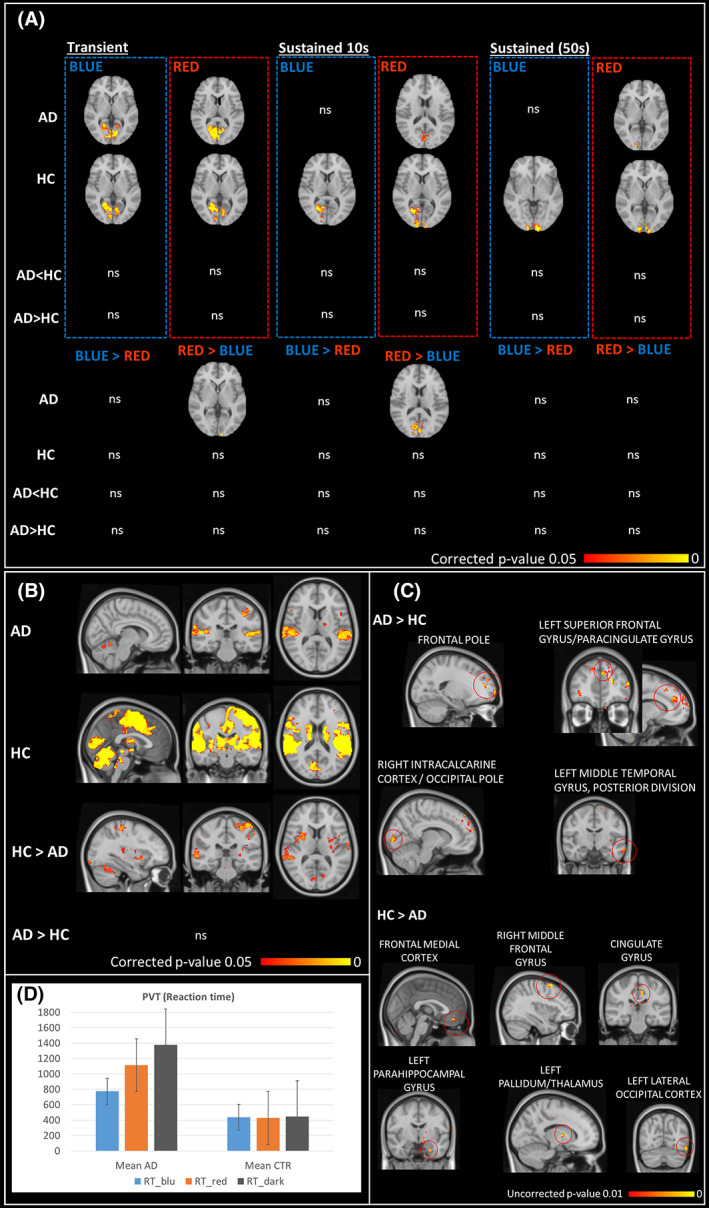
Functional brain MRI activations results. Panel A: functional brain responses to blue and red light stimulations for transient and sustained stimuli. Panel B: brain responses to the cognitive sustained attention task. Panel C: functional brain activations for the comparison between PVT task performed under blue light stimulation compared to PVT performed under red light stimulation (PVTblue > PVTred). Panel D: PVT reaction time under the different light conditions.

Control subjects showed primary occipital cortex activation in response to both transient and sustained (10 s and 50 s) stimuli for both red and blue light without significant differences between the two light conditions. Instead, AD patients showed reduced cortical activations to blue light compared to red light in response to transient and sustained (10 s) stimuli. Moreover, when considering only activations under blue light, they did not show significant responses to sustained stimuli (10 s and 50 s) (Fig. 6, panel A). In the AD group, the reduced cortical activations to blue light compared to red light in response to transient stimuli (blue < red) showed significant positive correlations with disease duration (*r* = 0.689; *p* = 0.013) and negative correlations with the inferior RNFL thickness at OCT (*r* = 0.806; *p* = 0.008). Instead, in the control group, the sustained response to blue light positively correlated with some actigraphic parameters (i.e., RA, *r* = 0.606; *p* = 0.029 and SE, *r* = 0.589; *p* = 0.042).

### Cognitive activation

Cerebral responses to PVT, in both groups, involved brain regions associated with sustained attention; however, these responses were significantly reduced in AD patients compared to controls within occipital, temporal, insular, parietal, and cerebellar cortices (Fig. 6, panel B).

Reaction time in AD was overall longer compared to controls even though this difference was not significant due to large variability of responses, especially in the AD group. Moreover, the lower brain responses for AD compared to controls correlate with performance obtained in different cognitive tasks, such as MMSEc, those evaluating executive functions, memory and language abilities (*p* < 0.001).

### Visual and cognitive stimuli interaction

The effect of blue light compared to red light on brain responses during the cognitive task was more evident in cerebral regions involved in visual functions (such the intracalcarine and occipital lateral cortex) and in brain regions crucial for vigilance and sustained attention (such as the frontal pole, superior and middle frontal gyri, middle temporal gyrus) (Fig. 6, panel C). Even if not statistically significant, in AD patients there seem to be a tendency toward an improvement of the cognitive performances under light stimulation in particular with blue light ([mean ± SD], median, AD: RT_blue_ [775 ± 784, 444] ms, RT_red_ [1113 ± 1813, 468] ms, RT_dark_ [1376 ± 2293, 532] ms; HC: RT_blue_ [438 ± 118, 432] ms, RT_red_ [427 ± 116, 419], RT_dark_ [447 ± 132, 455] ms) (Fig. 6, panel D).

Brain activations for the contrast PVT_blue_ > PVT_red_ in the comparison HC > AD the cingulate gyrus correlate with the SE (*r* = −0.776; *p* = 0.005) in AD patients.

## Discussion

In this study, we evaluated in vivo the mRGC‐system through a multimodal approach including OCT, actigraphy, chromatic pupillometry, and fMRI in a cohort of mild–moderate AD patients demonstrating that mRGCs are affected in AD, as highlighted by actigraphic, pupillometry, and fMRI findings. Each approach provides a specific layer of information that deserves a comment.

First, the OCT analysis failed to show significant differences between AD and controls except for the infero‐temporal GCL thickness. This sector corresponds to the supero‐nasal sector of the optic nerve, in line with previous results pointing to a preferential loss of the magnocellular component of the optic nerve in AD.^15^, ^19^, ^46^ Thus, the absence of significant RNFL differences suggests that in this group of mild AD with short disease duration an early pathology of the RGC cell body may occur before axonal damage becomes evident. Differently, in another cohort of AD, we have previously shown a significant thinning of the RNFL, mostly in the superior sector of the optic nerve.^18^ This discrepancy is most probably explained by the longer disease duration and the greater disease severity of this AD cohort and different OCT technologies used.^18^ Similarly to our results, other OCT studies failed to document significant differences in terms of RNFL between AD and controls.^47^, ^48^ We also found a significant decline of the average GCL thickness by age in both control and AD groups, which, remarkably, in controls was always significant between the 50–59 decade as compared to all the other decades, whereas in AD only in the comparison between the decades 50–59 and over 80s. Thus, in controls there is a major age‐related decline starting after 60 years of age, whereas in AD this is already evident in the 50–59 decade. In general, however, OCT measurements are age and disease severity‐related and only indirectly may be linked to mRGC pathology, if we consider that mRGCs represent only about 1% of total RGCs.

Second, the actigraphic recordings showed a significant difference between controls and AD for the TST, TIB, SL, and TAS. The increased TST found in the AD group is in line with previous findings.^49^ However, considering the circadian parameters (RA, IS, and IV), the two groups did not significantly differ even though the presence of a subgroup of circadian‐impaired AD patients was evident, as previously reported.^18^ We also demonstrated a decline of actigraphic parameters by age in both controls and AD for RA, L5, and TAS. Overall, actigraphic results confirm the presence of circadian and sleep dysfunction in at least a subgroup of AD patients at early stage of disease, and these abnormalities worsened with aging. Remarkably, when we evaluated the circadian parameters in the 3 centenarians, we found that some of the sleep parameters were better in centenarians than in AD patients in the 80–90 decade, as well as we observed the absence of significant worsening in centenarians in comparison with the controls from the same decade. This supports the concept of a successful aging in this group of super‐controls, which may be contributed by the maintenance of circadian synchronization possibly due to mRGC‐dependent circadian photoentrainment.^50^

Third, in relation to pupillometry studies, we recently published a preliminary study demonstrating a significant reduction of the peak amplitude for the rod‐condition in AD compared to controls as well as a significant correlation with age only in the AD group and a significant increase of the PIPR variance in the melanopsin protocol in the same AD patients here investigated.^23^ We interpreted these results, considering the retinal circuitry connecting mRGCs with rods,^51^ as a sign of ongoing pathology affecting mRGC dendrites, thus the connections between mRGCs with classical photoreceptors,^23^ as supported by the occurrence of dendropathy affecting mRGCs in *post mortem* AD retinas.^18^ Overall, these findings are compatible with mRGC pathology that does not affect yet the cell body but only the dendritic connections.^51^ In the current study, using a curve fitting approach we calculated the contraction onset timing and the average slope of the PLR, and using the exponential fitting of the constriction phase of the PLR we calculated lambda and |B| parameters of the PLR. Under rod‐condition, AD patients showed a significantly delayed onset of transient PLR response as well as a significantly lower average slope, and also a significantly reduced |B| indicative of a lower exponential decay amplitude. These pupillometric results overall reinforce and are in line with our previous findings^23^ again pointing toward an early dendropathy affecting mRGCs. In the 3 centenarians, we found a significant reduction only of the PIPR, which might suggest a loss of mRGCs with aging, as previously reported.^14^, ^52^

The significant correlation emerging between pupillometric and actigraphic measurements in controls argues in favor of a cross‐validity of these two approaches in capturing the mRGC‐mediated non‐visual functions, further validating the sensitivity of these tools to explore the mRGC system in vivo.

As final approach, brain fMRI results revealed reduced occipital cortex responses to blue light compared to red light stimulation in AD compared to controls, in particular for the sustained responses to light. This result further complements in vivo the mRGC loss previously reported in *post mortem* studies,^18^ as well as the pupillometry and actigraphic results.^23^ Interestingly, the reduced brain response to blue light compared to red light is correlated with disease duration in AD. As expected, the activation of brain areas is significantly lower in the psychomotor vigilance test in AD patients, mainly within temporal, insular, parietal, and cerebellar cortices, and the performances are worse in AD. When the cognitive task is combined with the light stimulation, performances in AD patients tend to be slightly better under the blue light and this stimulation is associated with the activation of not only brain areas involved in visual processing but also brain areas involved in vigilance and sustained attention, such as medial frontal cortex, medial frontal gyrus, and cingulate gyrus, where the activation is reduced compared to controls. Taken together, these findings might support the role of mRGCs in mediating brain cognitive responses, as previously described in healthy subjects and Leber's hereditary optic neuropathy patients.^24^, ^29^ At the same time occipital, frontal and temporal regions show higher brain activity under blue versus red light stimulation in AD compared to controls, indicating a possible compensatory effect.

Overall, the findings of our multimodal approach point to a dysfunctional mRGC system since the early stages of AD. Considering also the results of previous studies, we envisage a specific pathology affecting the mRGC cell body, reflected at initial stages by the dendropathy involving the connections with rods, detected by pupillometry, as well as by the reduction of the GCL thickness. Only at later stages, this mRGC pathology evolves into axonal damage, included in the general RNFL thinning. The dysfunction of the mRGC system ultimately contributes to the well‐known circadian and sleep disturbances reported in AD, documented by actigraphic studies. The timing of this process may vary in subgroups of AD patients, as reflected in the great variability observed for the multiple readouts that we used in our multimodal approach. Our findings may also support the use of light therapy as a counteracting measure for circadian dysfunction in AD aimed at stimulating the still functional mRGCs.^53^ Finally, mRGC function, as studied in vivo by multiple approaches providing different layers of information, may be proposed as a potential biomarker useful for stratifying the risk of disease conversion from MCI to AD and to identify subgroup of AD patients who are at increased risk to develop circadian and sleep disturbances.

This study has some limitations which include the limited number of patients enrolled, the absence of complete data for all of the patients examined and the inclusion of mild cases, which was necessary for compliance in running the tests.

In conclusion, the use of such a multimodal approach for investigating the mRGC system, particularly in neurodegenerative disorders characterized by circadian and sleep dysfunction, is here proposed as an effective tool for evaluating in vivo the mRGC‐dependent occurrence of circadian dysfunction, and biomarking patients for risk stratification.

## Author Contributions

CL, MM, RL, and VC contributed to the conception and design of the study; CL, MM, MR, MSM, SE, MD, SC, CB, CT, GV, MC, PD, MF, PA, CZ, and FB contributed to the acquisition and analysis of data; CL, MM, MR, SE, SC, CT, GV, AS, MF, PA, PB, RL, CT, RL, and VC contributed to drafting the text or preparing the figures.

## Conflict of Interest Statement

The authors declare no conflicts of interest related to this manuscript.

## Supporting information


**Table S1**. Spearman correlations between actigraphic and pupillometric variables in controls.
**Table S2**. Spearman correlations between actigraphic and pupillometric variables in AD.

## Data Availability

For original data, please contact Chiara La Morgia (chiara.lamorgia@unibo.it).

## References

[1] Hannibal J , Hindersson P , Knudsen SM , Georg B , Fahrenkrug J . The photopigment melanopsin is exclusively present in pituitary adenylate cyclase‐activating polypeptide‐containing retinal ganglion cells of the retinohypothalamic tract. J Neurosci. 2002;22(1):RC191. 11756521 10.1523/JNEUROSCI.22-01-j0002.2002 PMC6757615

[2] Berson DM , Dunn FA , Takao M . Phototransduction by retinal ganglion cells that set the circadian clock. Science. 2002;295(5557):1070‐1073. 11834835 10.1126/science.1067262

[3] Hattar S , Liao HW , Takao M , Berson DM , Yau KW . Melanopsin‐containing retinal ganglion cells: architecture, projections, and intrinsic photosensitivity. Science. 2002;295(5557):1065‐1070. 11834834 10.1126/science.1069609 PMC2885915

[4] Aranda ML , Schmidt TM . Diversity of intrinsically photosensitive retinal ganglion cells: circuits and functions. Cell Mol Life Sci. 2021;78(3):889‐907. 32965515 10.1007/s00018-020-03641-5 PMC8650628

[5] Oosterman JM , van Someren EJ , Vogels RL , Van Harten B , Scherder EJ . Fragmentation of the rest‐activity rhythm correlates with age‐related cognitive deficits. J Sleep Res. 2009;18(1):129‐135. 19250179 10.1111/j.1365-2869.2008.00704.x

[6] Wu YH , Swaab DF . Disturbance and strategies for reactivation of the circadian rhythm system in aging and Alzheimer's disease. Sleep Med. 2007;8(6):623‐636. 17383938 10.1016/j.sleep.2006.11.010

[7] Froy O . Circadian rhythms, aging, and life span in mammals. Physiology (Bethesda). 2011;26(4):225‐235. 21841071 10.1152/physiol.00012.2011

[8] Li P , Gao L , Gaba A , et al. Circadian disturbances in Alzheimer's disease progression: a prospective observational cohort study of community‐based older adults. Lancet Healthy Longev. 2020;1(3):e96‐e105. 34179863 10.1016/s2666-7568(20)30015-5 PMC8232345

[9] Ozcan GG , Lim S , Leighton P , Allison WT , Rihel J . Sleep is bi‐directionally modified by amyloid beta oligomers. eLife. 2020;9:e53995. 32660691 10.7554/eLife.53995 PMC7360368

[10] Tranah GJ , Blackwell T , Stone KL , et al. Circadian activity rhythms and risk of incident dementia and mild cognitive impairment in older women. Ann Neurol. 2011;70(5):722‐732. 22162057 10.1002/ana.22468 PMC3244839

[11] Do MTH . Melanopsin and the intrinsically photosensitive retinal ganglion cells: biophysics to behavior. Neuron. 2019;104(2):205‐226. 31647894 10.1016/j.neuron.2019.07.016 PMC6944442

[12] Johnson BM , Miao M , Sadun AA . Age‐related decline of human optic nerve axon populations. Age. 1987;10(1):5‐9.

[13] Feuer WJ , Budenz DL , Anderson DR , et al. Topographic differences in the age‐related changes in the retinal nerve fiber layer of normal eyes measured by stratus optical coherence tomography. J Glaucoma. 2011;20(3):133‐138. 20577113 10.1097/IJG.0b013e3181e079b2 PMC2946985

[14] La Morgia C , Ross‐Cisneros FN , Sadun AA , et al. Melanopsin retinal ganglion cells are resistant to neurodegeneration in mitochondrial optic neuropathies. Brain. 2010;133(Pt 8):2426‐2438. 20659957 10.1093/brain/awq155 PMC3139936

[15] Hinton DR , Sadun AA , Blanks JC , Miller CA . Optic‐nerve degeneration in Alzheimer's disease. N Engl J Med. 1986;315(8):485‐487. 3736630 10.1056/NEJM198608213150804

[16] Harper DG , Stopa EG , Kuo‐Leblanc V , et al. Dorsomedial SCN neuronal subpopulations subserve different functions in human dementia. Brain. 2008;131(Pt 6):1609‐1617. 18372313 10.1093/brain/awn049 PMC3286014

[17] Chan VTT , Sun Z , Tang S , et al. Spectral‐domain OCT measurements in Alzheimer's disease: a systematic review and meta‐analysis. Ophthalmology. 2019;126(4):497‐510. 30114417 10.1016/j.ophtha.2018.08.009 PMC6424641

[18] La Morgia C , Ross‐Cisneros FN , Koronyo Y , et al. Melanopsin retinal ganglion cell loss in Alzheimer disease. Ann Neurol. 2016;79(1):90‐109. 26505992 10.1002/ana.24548 PMC4737313

[19] Koronyo Y , Biggs D , Barron E , et al. Retinal amyloid pathology and proof‐of‐concept imaging trial in Alzheimer's disease. JCI Insight. 2017;2(16):e93621. 28814675 10.1172/jci.insight.93621 PMC5621887

[20] La Morgia C , Carelli V , Carbonelli M . Melanopsin retinal ganglion cells and pupil: clinical implications for neuro‐ophthalmology. Front Neurol. 2018;9:1047. 30581410 10.3389/fneur.2018.01047 PMC6292931

[21] La Morgia C , Romagnoli M , Pizza F , et al. Chromatic pupillometry in isolated rapid eye movement sleep behavior disorder. Mov Disord. 2022;37(1):205‐210. 34617633 10.1002/mds.28809 PMC9293298

[22] Oh AJ , Amore G , Sultan W , et al. Pupillometry evaluation of melanopsin retinal ganglion cell function and sleep‐wake activity in pre‐symptomatic Alzheimer's disease. PLoS ONE. 2019;14(12):e0226197. 31821378 10.1371/journal.pone.0226197 PMC6903762

[23] Romagnoli M , Stanzani Maserati M , De Matteis M , et al. Chromatic pupillometry findings in Alzheimer's disease. Front Neurosci. 2020;14:780. 32848556 10.3389/fnins.2020.00780 PMC7431959

[24] Vandewalle G , Schmidt C , Albouy G , et al. Brain responses to violet, blue, and green monochromatic light exposures in humans: prominent role of blue light and the brainstem. PLoS ONE. 2007;2(11):e1247. 18043754 10.1371/journal.pone.0001247 PMC2082413

[25] Vandewalle G , Maquet P , Dijk DJ . Light as a modulator of cognitive brain function. Trends Cogn Sci. 2009;13(10):429‐438. 19748817 10.1016/j.tics.2009.07.004

[26] Vandewalle G , Archer SN , Wuillaume C , et al. Effects of light on cognitive brain responses depend on circadian phase and sleep homeostasis. J Biol Rhythms. 2011;26(3):249‐259. 21628552 10.1177/0748730411401736

[27] Vandewalle G , Collignon O , Hull JT , et al. Blue light stimulates cognitive brain activity in visually blind individuals. J Cogn Neurosci. 2013;25(12):2072‐2085. 23859643 10.1162/jocn_a_00450 PMC4497579

[28] Daneault V , Hebert M , Albouy G , et al. Aging reduces the stimulating effect of blue light on cognitive brain functions. Sleep. 2014;37(1):85‐96. 24381372 10.5665/sleep.3314 PMC3865352

[29] Evangelisti S , La Morgia C , Testa C , et al. Brain functional MRI responses to blue light stimulation in Leber's hereditary optic neuropathy. Biochem Pharmacol. 2021;191:114488. 33647261 10.1016/j.bcp.2021.114488

[30] Perneczky R , Wagenpfeil S , Komossa K , Grimmer T , Diehl J , Kurz A . Mapping scores onto stages: mini‐mental state examination and clinical dementia rating. Am J Geriatr Psychiatry. 2006;14(2):139‐144. 16473978 10.1097/01.JGP.0000192478.82189.a8

[31] Dubois B , Feldman HH , Jacova C , et al. Advancing research diagnostic criteria for Alzheimer's disease: the IWG‐2 criteria. Lancet Neurol. 2014;13(6):614‐629. 24849862 10.1016/S1474-4422(14)70090-0

[32] Beck AT , Ward CH , Mendelson M , Mock J , Erbaugh J . An inventory for measuring depression. Arch Gen Psychiatry. 1961;4:561‐571. 13688369 10.1001/archpsyc.1961.01710120031004

[33] Spielberger CDVP , Barker LR , Donham GW , Westberry LG . The factor structure of the state‐trait anxiety inventory. In: Sarason IG , Spielberger CD , eds. Stress and Anxiety. Vol 7. Hemisphere Publishing; 1980:95‐109.

[34] Folstein MF , Folstein SE , McHugh PR . “Mini‐mental state”: a practical method for grading the cognitive state of patients for the clinician. J Psychiatr Res. 1975;12(3):189‐198. 1202204 10.1016/0022-3956(75)90026-6

[35] Gallassi R , Lenzi P , Stracciari A , et al. Neuropsychological assessment of mental deterioration: purpose of a brief battery and a probabilistic definition of “normality” and “non‐normality”. Acta Psychiatr Scand. 1986;74(1):62‐67. 3766185 10.1111/j.1600-0447.1986.tb06228.x

[36] Gallassi R , Oppi F , Poda R , et al. Are subjective cognitive complaints a risk factor for dementia? Neurol Sci. 2010;31(3):327‐336. 20182898 10.1007/s10072-010-0224-6

[37] van Someren EJ , Hagebeuk EE , Lijzenga C , et al. Circadian rest‐activity rhythm disturbances in Alzheimer's disease. Biol Psychiatry. 1996;40(4):259‐270. 8871772 10.1016/0006-3223(95)00370-3

[38] Goncalves BS , Adamowicz T , Louzada FM , Moreno CR , Araujo JF . A fresh look at the use of nonparametric analysis in actimetry. Sleep Med Rev. 2015;20:84‐91. 25065908 10.1016/j.smrv.2014.06.002

[39] Wang J , Xian H , Licis A , et al. Measuring the impact of apnea and obesity on circadian activity patterns using functional linear modeling of actigraphy data. J Circadian Rhythms. 2011;9(1):11. 21995417 10.1186/1740-3391-9-11 PMC3245508

[40] Drummond SP , Bischoff‐Grethe A , Dinges DF , Ayalon L , Mednick SC , Meloy MJ . The neural basis of the psychomotor vigilance task. Sleep. 2005;28(9):1059‐1068. 16268374

[41] Armstrong RA . Statistical guidelines for the analysis of data obtained from one or both eyes. Ophthalmic Physiol Opt. 2013;33(1):7‐14. 23252852 10.1111/opo.12009

[42] Ying GS , Maguire MG , Glynn R , Rosner B . Tutorial on biostatistics: linear regression analysis of continuous correlated eye data. Ophthalmic Epidemiol. 2017;24(2):130‐140. 28102741 10.1080/09286586.2016.1259636 PMC5597052

[43] Rosner B , Glynn RJ , Lee ML . Extension of the rank sum test for clustered data: two‐group comparisons with group membership defined at the subunit level. Biometrics. 2006;62(4):1251‐1259. 17156300 10.1111/j.1541-0420.2006.00582.x

[44] Eickhoff SB , Paus T , Caspers S , et al. Assignment of functional activations to probabilistic cytoarchitectonic areas revisited. Neuroimage. 2007;36(3):511‐521. 17499520 10.1016/j.neuroimage.2007.03.060

[45] Hatfield CF , Herbert J , van Someren EJ , Hodges JR , Hastings MH . Disrupted daily activity/rest cycles in relation to daily cortisol rhythms of home‐dwelling patients with early Alzheimer's dementia. Brain. 2004;127(Pt 5):1061‐1074. 14998915 10.1093/brain/awh129

[46] La Morgia C , Di Vito L , Carelli V , Carbonelli M . Patterns of retinal ganglion cell damage in neurodegenerative disorders: parvocellular vs magnocellular degeneration in optical coherence tomography studies. Front Neurol. 2017;8:710. 29312131 10.3389/fneur.2017.00710 PMC5744067

[47] Sanchez D , Castilla‐Marti M , Rodriguez‐Gomez O , et al. Usefulness of peripapillary nerve fiber layer thickness assessed by optical coherence tomography as a biomarker for Alzheimer's disease. Sci Rep. 2018;8(1):16345. 30397251 10.1038/s41598-018-34577-3 PMC6218495

[48] den Haan J , Verbraak FD , Visser PJ , Bouwman FH . Retinal thickness in Alzheimer's disease: a systematic review and meta‐analysis. Alzheimers Dement (Amst). 2017;6:162‐170. 28275698 10.1016/j.dadm.2016.12.014 PMC5328759

[49] Hooghiemstra AM , Eggermont LH , Scheltens P , van der Flier WM , Scherder EJ . The rest‐activity rhythm and physical activity in early‐onset dementia. Alzheimer Dis Assoc Disord. 2015;29(1):45‐49. 24632989 10.1097/WAD.0000000000000037

[50] Shen J , Tower J . Effects of light on aging and longevity. Ageing Res Rev. 2019;53:100913. 31154014 10.1016/j.arr.2019.100913 PMC6663583

[51] Hannibal J , Christiansen AT , Heegaard S , Fahrenkrug J , Kiilgaard JF . Melanopsin expressing human retinal ganglion cells: subtypes, distribution, and intraretinal connectivity. J Comp Neurol. 2017;525(8):1934‐1961. 28160289 10.1002/cne.24181

[52] Esquiva G , Lax P , Perez‐Santonja JJ , Garcia‐Fernandez JM , Cuenca N . Loss of melanopsin‐expressing ganglion cell subtypes and dendritic degeneration in the aging human retina. Front Aging Neurosci. 2017;9:79. 28420980 10.3389/fnagi.2017.00079 PMC5378720

[53] Mitolo M , Tonon C , La Morgia C , Testa C , Carelli V , Lodi R . Effects of light treatment on sleep, cognition, mood, and behavior in Alzheimer's disease: a systematic review. Dement Geriatr Cogn Disord. 2018;46(5–6):371‐384. 30537760 10.1159/000494921

