# Supplementary material for: Multimodal investigation of melanopsin retinal ganglion cells in Alzheimer's disease
Source: Ann Clin Transl Neurol. 2023 Apr 23;10(6):918–32. doi: 10.1002/acn3.51773 (PMC10270274; doi:10.1002/acn3.51773)
Supplement: Supplementary file 1 — Table S1. Spearman correlations between actigraphic and pupillometric variables in controls. Table S2. Spearman correlations between actigraphic and pupillometric variables in AD. [file ACN3-10-918-s001.docx]

**Supplementary Table 1.** Spearman correlations between actigraphic and pupillometric variables in controls.

| **Variable 1** | **Variable 2** | **rho** | **95% CI** | | **p-value** | **p-value _adjusted_** |
| --- | --- | --- | --- | --- | --- | --- |
|  |  |  | **LL** | **UL** |  |  |
| RA | Contraction Onset Timing | -0.84 | -0.94 | -0.60 | 2.17E-05 | 0.0010 |
| M10 | Slope | 0.83 | 0.57 | 0.94 | 4.13E-05 | 0.0020 |
| RA | \|B\| | 0.70 | 0.32 | 0.89 | 0.001719 | 0.0825 |
| RA | Lambda | -0.69 | -0.88 | -0.29 | 0.002349 | 0.1128 |
| FI | Contraction Onset Timing | 0.66 | 0.24 | 0.87 | 0.004209 | 0.2020 |
| L5 | Lambda | 0.61 | 0.16 | 0.85 | 0.00964 | 0.4627 |
| FI | Lambda | 0.59 | 0.13 | 0.84 | 0.013408 | 0.6436 |
| AWT | Contraction Onset Timing | 0.58 | 0.13 | 0.84 | 0.01418 | 0.6806 |
| FI | \|B\| | -0.55 | -0.82 | -0.08 | 0.022003 | 1.0000 |
| TAS | Contraction Onset Timing | 0.53 | 0.06 | 0.81 | 0.027533 | 1.0000 |
| AWT | Lambda | 0.52 | 0.04 | 0.81 | 0.030654 | 1.0000 |
| L5 | Contraction Onset Timing | 0.46 | -0.05 | 0.78 | 0.065148 | 1.0000 |
| SE | Lambda | -0.45 | -0.77 | 0.05 | 0.06653 | 1.0000 |
| TAS | Lambda | 0.44 | -0.06 | 0.77 | 0.074468 | 1.0000 |
| AWT | \|B\| | -0.43 | -0.76 | 0.08 | 0.087805 | 1.0000 |
| SE | Contraction Onset Timing | -0.42 | -0.75 | 0.10 | 0.097197 | 1.0000 |
| TIB | Contraction Onset Timing | 0.41 | -0.10 | 0.75 | 0.101166 | 1.0000 |
| IV | Contraction Onset Timing | 0.40 | -0.11 | 0.75 | 0.10854 | 1.0000 |
| M10 | Contraction Onset Timing | -0.40 | -0.74 | 0.12 | 0.116023 | 1.0000 |
| IS | Slope | 0.38 | -0.14 | 0.73 | 0.136949 | 1.0000 |
| TIB | \|B\| | -0.37 | -0.73 | 0.15 | 0.144313 | 1.0000 |
| M10 | \|B\| | 0.36 | -0.16 | 0.73 | 0.15243 | 1.0000 |
| L5 | \|B\| | -0.35 | -0.72 | 0.17 | 0.171013 | 1.0000 |
| TST | Slope | -0.34 | -0.71 | 0.18 | 0.178447 | 1.0000 |
| TIB | Lambda | 0.34 | -0.18 | 0.71 | 0.181669 | 1.0000 |
| TAS | Slope | 0.33 | -0.20 | 0.71 | 0.201129 | 1.0000 |
| IV | \|B\| | -0.32 | -0.70 | 0.20 | 0.203143 | 1.0000 |
| IV | Lambda | 0.32 | -0.20 | 0.70 | 0.210444 | 1.0000 |
| IS | \|B\| | 0.31 | -0.21 | 0.70 | 0.220104 | 1.0000 |
| L5 | Slope | 0.31 | -0.22 | 0.69 | 0.231525 | 1.0000 |
| SL | Contraction Onset Timing | 0.28 | -0.25 | 0.68 | 0.275279 | 1.0000 |
| SE | \|B\| | 0.28 | -0.25 | 0.68 | 0.281536 | 1.0000 |
| TIB | Slope | -0.26 | -0.67 | 0.27 | 0.312721 | 1.0000 |
| IS | Contraction Onset Timing | -0.25 | -0.66 | 0.28 | 0.331943 | 1.0000 |
| SE | Slope | -0.24 | -0.66 | 0.28 | 0.345353 | 1.0000 |
| TST | Contraction Onset Timing | 0.21 | -0.32 | 0.63 | 0.426754 | 1.0000 |
| IS | Lambda | -0.20 | -0.63 | 0.32 | 0.439234 | 1.0000 |
| SL | \|B\| | -0.17 | -0.61 | 0.35 | 0.518725 | 1.0000 |
| SL | Lambda | 0.15 | -0.37 | 0.60 | 0.553293 | 1.0000 |
| TAS | \|B\| | -0.14 | -0.59 | 0.38 | 0.586263 | 1.0000 |
| RA | Slope | 0.13 | -0.38 | 0.59 | 0.611478 | 1.0000 |
| TST | \|B\| | -0.12 | -0.58 | 0.40 | 0.646143 | 1.0000 |
| M10 | Lambda | 0.09 | -0.42 | 0.56 | 0.736305 | 1.0000 |
| SL | Slope | -0.06 | -0.54 | 0.45 | 0.818206 | 1.0000 |
| AWT | Slope | 0.04 | -0.46 | 0.53 | 0.864831 | 1.0000 |
| IV | Slope | -0.02 | -0.51 | 0.47 | 0.924635 | 1.0000 |
| TST | Lambda | 0.01 | -0.49 | 0.50 | 0.977656 | 1.0000 |
| FI | Slope | -0.01 | -0.50 | 0.49 | 820.559 | 1.0000 |

Abbreviations

RA=relative amplitude; IS=interdaily stability; IV= intradaily variability; M10=Most 10 average, activity during most 10 active hours; L5=Least 5 average, activity for least 5 active hours; SE (Sleep Efficiency); TST (Total Sleep Time); TIB (Time In Bed); AWT (Actual Wake Time); SL (Sleep Latency); TAS (Total Activity Score); FI (Fragmentation Index);

LL= lower limit; UL= upper limit.

**Supplementary Table 2.** Spearman correlations between actigraphic and pupillometric variables in AD.

| **Parameter 1** | **Parameter 2** | **rho** | **95% CI** | | **p-value** | **p-value _adjusted_** |
| --- | --- | --- | --- | --- | --- | --- |
|  |  |  | **LL** | **UL** |  |  |
| TIB | \|B\| | -0.65 | -0.84 | -0.32 | 0.000597 | 0.048 |
| IS | Slope | -0.62 | -0.82 | -0.28 | 0.001135 | 0.091 |
| TIB | Slope | -0.54 | -0.78 | -0.16 | 0.00643 | 0.514 |
| TIB | Contraction Onset Timing | 0.52 | 0.14 | 0.77 | 0.009192 | 0.735 |
| TST | \|B\| | -0.50 | -0.76 | -0.11 | 0.013399 | 1.000 |
| TST | Slope | -0.45 | -0.73 | -0.04 | 0.028737 | 1.000 |
| RA | disease duration | -0.43 | -0.71 | -0.04 | 0.028777 | 1.000 |
| IS | MMSEc | -0.42 | -0.70 | -0.03 | 0.031685 | 1.000 |
| RA | Slope | -0.42 | -0.71 | -0.01 | 0.039592 | 1.000 |
| TST | Contraction Onset Timing | 0.41 | 0.00 | 0.70 | 0.046656 | 1.000 |
| disease duration | \|B\| | 0.41 | -0.01 | 0.70 | 0.047142 | 1.000 |
| SL | Slope | -0.39 | -0.69 | 0.03 | 0.060324 | 1.000 |
| RA | MMSEc | -0.34 | -0.65 | 0.07 | 0.089572 | 1.000 |
| TIB | MMSEc | -0.33 | -0.65 | 0.07 | 0.09604 | 1.000 |
| SL | \|B\| | -0.35 | -0.67 | 0.08 | 0.09618 | 1.000 |
| L5 | Lambda | -0.35 | -0.67 | 0.08 | 0.097072 | 1.000 |
| disease duration | Lambda | -0.34 | -0.66 | 0.09 | 0.103291 | 1.000 |
| IS | \|B\| | -0.34 | -0.66 | 0.09 | 0.103488 | 1.000 |
| M10 | Slope | -0.34 | -0.66 | 0.09 | 0.104485 | 1.000 |
| IV | Slope | 0.34 | -0.09 | 0.66 | 0.104884 | 1.000 |
| L5 | disease duration | 0.32 | -0.09 | 0.64 | 0.10794 | 1.000 |
| MMSEc | \|B\| | 0.31 | -0.12 | 0.64 | 0.146093 | 1.000 |
| MMSEc | Slope | 0.30 | -0.13 | 0.63 | 0.156632 | 1.000 |
| L5 | MMSEc | 0.28 | -0.13 | 0.61 | 0.165897 | 1.000 |
| L5 | Slope | 0.28 | -0.16 | 0.62 | 0.19196 | 1.000 |
| RA | \|B\| | -0.27 | -0.62 | 0.16 | 0.199711 | 1.000 |
| disease duration | Slope | 0.27 | -0.17 | 0.61 | 0.208628 | 1.000 |
| L5 | \|B\| | 0.26 | -0.17 | 0.61 | 0.21893 | 1.000 |
| TIB | Lambda | 0.26 | -0.18 | 0.61 | 0.228319 | 1.000 |
| IV | disease duration | 0.24 | -0.18 | 0.58 | 0.243927 | 1.000 |
| FI | Contraction Onset Timing | 0.24 | -0.19 | 0.59 | 0.258219 | 1.000 |
| RA | Lambda | 0.24 | -0.19 | 0.59 | 0.260428 | 1.000 |
| M10 | Lambda | -0.24 | -0.59 | 0.20 | 0.26582 | 1.000 |
| SL | Contraction Onset Timing | 0.23 | -0.21 | 0.59 | 0.288037 | 1.000 |
| TST | Lambda | 0.23 | -0.21 | 0.59 | 0.2881 | 1.000 |
| TST | MMSEc | -0.21 | -0.56 | 0.20 | 0.297506 | 1.000 |
| FI | \|B\| | -0.21 | -0.57 | 0.22 | 0.321605 | 1.000 |
| SE | \|B\| | 0.21 | -0.23 | 0.57 | 0.334908 | 1.000 |
| TAS | disease duration | 0.19 | -0.22 | 0.55 | 0.347204 | 1.000 |
| SL | disease duration | 0.19 | -0.22 | 0.55 | 0.349559 | 1.000 |
| TAS | \|B\| | -0.20 | -0.57 | 0.23 | 0.353049 | 1.000 |
| SE | Contraction Onset Timing | -0.20 | -0.57 | 0.24 | 0.354674 | 1.000 |
| AWT | Contraction Onset Timing | 0.17 | -0.26 | 0.55 | 0.419517 | 1.000 |
| AWT | disease duration | 0.16 | -0.26 | 0.52 | 0.439344 | 1.000 |
| IV | \|B\| | 0.16 | -0.27 | 0.54 | 0.447654 | 1.000 |
| AWT | \|B\| | -0.16 | -0.54 | 0.28 | 0.467671 | 1.000 |
| IS | disease duration | -0.15 | -0.51 | 0.27 | 0.477985 | 1.000 |
| MMSEc | Contraction Onset Timing | 0.15 | -0.28 | 0.53 | 0.481587 | 1.000 |
| TAS | MMSEc | -0.14 | -0.51 | 0.28 | 0.502948 | 1.000 |
| IS | Contraction Onset Timing | 0.14 | -0.29 | 0.52 | 0.516943 | 1.000 |
| M10 | MMSEc | -0.11 | -0.49 | 0.30 | 0.581842 | 1.000 |
| TAS | Contraction Onset Timing | 0.12 | -0.31 | 0.51 | 0.587342 | 1.000 |
| IV | Lambda | -0.10 | -0.50 | 0.33 | 0.634661 | 1.000 |
| SE | Slope | 0.09 | -0.34 | 0.49 | 0.678123 | 1.000 |
| SE | disease duration | -0.08 | -0.47 | 0.32 | 0.684609 | 1.000 |
| IS | Lambda | -0.09 | -0.48 | 0.34 | 0.69061 | 1.000 |
| M10 | disease duration | -0.08 | -0.46 | 0.33 | 0.694708 | 1.000 |
| FI | Slope | -0.08 | -0.48 | 0.34 | 0.694786 | 1.000 |
| FI | disease duration | 0.08 | -0.33 | 0.46 | 0.70734 | 1.000 |
| MMSEc | Lambda | -0.07 | -0.47 | 0.36 | 0.752679 | 1.000 |
| SE | MMSEc | 0.06 | -0.35 | 0.45 | 0.770143 | 1.000 |
| FI | Lambda | -0.05 | -0.46 | 0.37 | 0.805559 | 1.000 |
| IV | Contraction Onset Timing | 0.05 | -0.37 | 0.46 | 0.810051 | 1.000 |
| RA | Contraction Onset Timing | -0.05 | -0.45 | 0.37 | 0.824227 | 1.000 |
| SL | Lambda | -0.04 | -0.45 | 0.38 | 0.841678 | 1.000 |
| L5 | Contraction Onset Timing | 0.04 | -0.38 | 0.44 | 0.870088 | 1.000 |
| SE | Lambda | -0.03 | -0.44 | 0.39 | 0.876561 | 1.000 |
| M10 | Contraction Onset Timing | -0.03 | -0.44 | 0.39 | 0.897216 | 1.000 |
| M10 | \|B\| | -0.03 | -0.43 | 0.39 | 0.90689 | 1.000 |
| AWT | Slope | 0.02 | -0.40 | 0.43 | 0.91875 | 1.000 |
| AWT | Lambda | 0.02 | -0.40 | 0.43 | 0.932507 | 1.000 |
| disease duration | Contraction Onset Timing | -0.02 | -0.43 | 0.40 | 0.937141 | 1.000 |
| TST | disease duration | -0.01 | -0.41 | 0.39 | 0.948604 | 1.000 |
| SL | MMSEc | -0.01 | -0.41 | 0.39 | 0.957648 | 1.000 |
| IV | MMSEc | 0.01 | -0.39 | 0.41 | 0.959652 | 1.000 |
| AWT | MMSEc | -0.01 | -0.41 | 0.39 | 0.962963 | 1.000 |
| TIB | disease duration | -0.01 | -0.40 | 0.39 | 0.969535 | 1.000 |
| FI | MMSEc | 0.01 | -0.39 | 0.40 | 0.969574 | 1.000 |
| TAS | Slope | 0.00 | -0.41 | 0.42 | 0.983725 | 1.000 |
| TAS | Lambda | 0.00 | -0.41 | 0.42 | 0.98713 | 1.000 |

Abbreviations

RA=relative amplitude; IS=interdaily stability; IV= intradaily variability; M10=Most 10 average, activity during most 10 active hours; L5=Least 5 average, activity for least 5 active hours; SE (Sleep Efficiency); TST (Total Sleep Time); TIB (Time In Bed); AWT (Actual Wake Time); SL (Sleep Latency); TAS (Total Activity Score); FI (Fragmentation Index);

LL= lower limit; UL= upper limit.
